# Novel adipokine associated with OA: retinol binding protein 4 (RBP4) is produced by cartilage and is correlated with MMPs in osteoarthritis patients

**DOI:** 10.1007/s00011-020-01326-0

**Published:** 2020-02-24

**Authors:** Morena Scotece, Anna Koskinen-Kolasa, Antti Pemmari, Tiina Leppänen, Mari Hämäläinen, Teemu Moilanen, Eeva Moilanen, Katriina Vuolteenaho

**Affiliations:** 1 The Immunopharmacology Research Group, Faculty of Medicine and Health Technology, Tampere University and Tampere University Hospital, 33014 Tampere, Finland; 2 Coxa Hospital for Joint Replacement, 33520 Tampere, Finland

**Keywords:** Adipokines, Chondrocytes, Cartilage, Matrix metalloproteinases, Osteoarthritis

## Abstract

**Objective:**

Retinol binding protein 4 (RBP4) is a member of the lipocalin family and a vitamin A carrier in the blood. More recently, RBP4 has been described as an adipokine that is involved in insulin resistance and metabolic syndrome (MetS). As obesity, MetS and some adipokines contribute to the pathogenesis of osteoarthritis (OA), we investigated RBP4 in patients with OA.

**Materials and methods:**

Cartilage, synovial fluid and blood samples were collected from 100 OA patients undergoing total knee replacement surgery. Primary chondrocytes and cartilage tissue were cultured to measure the RBP4 expression. The concentrations of RBP4, other adipokines (adipsin, adiponectin, leptin and resistin) and biomarkers of OA (COMP, MMP-1, MMP-3 and YKL-40) were measured by immunoassay, and gene expression was measured by next-generation RNA sequencing.

**Results:**

The OA cartilage samples released RBP4 into the culture medium, and the levels correlated positively with the expression of the adipokines adipsin, adiponectin, leptin and resistin. RBP4 was the most prominently expressed of these adipokines in the OA chondrocytes, and the expression of the RBP4 receptors STRA6 (stimulated by retinoic acid gene homologue 6) and TLR4 (Toll-like receptor 4) was also detected. Within the cartilage culture medium, RBP4 showed a positive correlation with MMP-1, MMP-3 and YKL-40. RBP4 was also present in the synovial fluid from the OA patients and correlated positively with the concentrations of RBP4 found in the plasma and the cartilage culture medium. Plasma RBP4 concentrations also showed a positive correlation with MMP-3 and adipsin.

**Conclusions:**

We show here, for the first time, that RBP4 is produced within OA joints and that it is associated with increased levels of adipokines and MMPs. The results suggest a role for RBP4 in the pathogenesis of OA and as a possible target for the disease-modifying drugs for the treatment of OA.

## Introduction

Osteoarthritis (OA) is the most prevalent joint disease and a leading cause of disability that affects an estimated 10% of the world’s population over the age of 60 years [1, 2]. It is a chronic disease that commonly affects the entire joint structure [3]. Degradation of the articular cartilage, formation of osteophytes, subchondral bone sclerosis and synovial inflammation are the principal changes in OA-affected joints. Accumulating evidence supports the contention that low-grade inflammation is critical to the pathogenesis of OA [4]. OA is related to ageing, but it is also associated with a variety of risk factors, including genetic predisposition, trauma, gender and, in particular, obesity.

Adipose tissue produces cytokine-like hormones known as ‘adipokines’. Adipokines not only regulate energy metabolism and appetite but also other functions in the human body. Several studies have reported an important role for adipokines in cartilage and bone homeostasis, metabolism and inflammation and suggest that these molecules serve as a link between obesity and OA [5]. Retinol binding protein 4 (RBP4) was first identified as an adipocyte-derived factor that contributes to the pathogenesis of type 2 diabetes [6]. RBP4 is most prevalently expressed in the liver, followed by adipose tissue [7]. In the circulation, RBP4 is the sole retinol (vitamin A) transport protein that moves vitamin A from the liver to the peripheral tissues where it is metabolized to retinoic acid [8]. Increased levels of RBP4 in obese and insulin-resistant humans and in mouse models have been reported [6, 9] as has a strong correlation of serum RBP4 levels with obesity and insulin resistance [10, 11]; however, these findings were not found in all studies [12, 13]. Several studies have also shown a correlation between RBP4 and other components of human metabolic syndrome (MetS), such as dyslipidaemia [14], hypertension [15] and cardiovascular diseases [16].

RBP4 acts by binding to the receptor ‘stimulated by retinoic acid gene homologue 6′ (STRA6) [17, 18]. In addition, some of its effects are mediated by Toll-like receptor 4 (TLR4) [19–21], which is a major pathway that induces the expression of inflammatory and catabolic factors in chondrocytes and other cells. Therefore, we aimed to investigate whether the adipokine RBP4 is associated with OA; we analysed RBP4 levels in the plasma, synovial fluid (SF) and cartilage from OA patients and determined the correlation of RBP4 with other adipokines and biomarkers implicated in OA.

## Materials and methods

### Subjects

One hundred OA patients [body mass index (BMI) 29.7 (8.3) kg/m^2^; age 72 (14) years, median (interquartile range, IQR); 62/38 females/males] undergoing total knee replacement surgery at Coxa Hospital for Joint Replacement, Tampere, Finland, participated in the study. All patients fulfilled the American College of Rheumatology classification criteria for OA [22]. The study was approved by the Ethics Committee of Tampere University Hospital, and the patients gave their written informed consent to participate in the study.

### Cartilage, synovial fluid and blood samples

The cartilage samples (*n* = 97) were processed as previously described [23] and cultured in Dulbecco’s modified Eagle’s medium (DMEM) with Glutamax I containing 10% heat-inactivated foetal bovine serum and penicillin (100 units/ml), streptomycin (100 µg/ml)***, and amphotericin B (250 ng/ml) (all obtained from Invitrogen Carlsbad, CA, USA). The cartilage samples were cultured in a six-well plate and after 42 h, the culture medium was collected and stored at − 20 °C.

Synovial fluid samples were collected by joint puncture at the beginning of the arthroplasty and centrifuged at 4000*g* for 15 min at 4 °C, and the supernatants were stored at − 70 °C until analysed. The SF samples were available from 68 OA patients for this study.

The blood samples were obtained from all patients just prior to their operation, and the plasma was separated by centrifugation at 1200 rpm for 10 min at 4 °C and stored at − 70 °C until analysed.

### Enzyme-linked immunosorbent assay (ELISA)

Concentrations of the adipokines RBP4, adipsin, adiponectin, leptin and resistin, as well as those of the OA biomarkers cartilage oligomeric matrix protein (COMP), matrix metalloproteinase 1 (MMP-1), matrix metalloproteinase 3 (MMP-3) and chitinase-3-like protein 1 (CHI3L1, also known as YKL-40), were measured by immunoassay (all obtained from R&D Systems Europe Ltd, Abingdon, UK, except COMP which was obtained from BioVendor Research and Diagnostic Products, Modřice, Czech Republic).

### Next-generation sequencing (NGS) and data analysis

Analysis of mRNA expression was performed using chondrocytes isolated from the knee cartilages from ten additional OA patients whose samples were not used in the experiments described above [*n* = 10; BMI 27.3 (5.8) kg/m^2^; age 70 (15) years, median (IQR); 4/6 females/males] undergoing knee replacement surgery at Coxa Hospital for Joint Replacement, Tampere, Finland. The cells were isolated as described [24] and cultured for 24 h. Total RNA was isolated, and next-generation sequencing (NGS) was carried out with Illumina HiSeq2500 according to the manufacturer’s instructions (Illumina Inc., CA, USA) at the Finnish Institute of Molecular Medicine (FIMM) sequencing core, Helsinki, Finland.

The sequencing depth was 20 million paired-end reads of 100 bp. The read quality was first assessed using FastQC [25], and the reads were trimmed using Trimmomatic [26]. The trimmed reads were aligned to the reference human genome with STAR [27]. The count matrices were prepared with the featureCounts program [28]. Gene counts were normalized with the DESeq2 method [29] implemented in the Chipster software package [30]. For each gene, a geometric mean of count values across all samples was calculated. Then, the count value in each sample was divided by this mean. For each sample, a median of values obtained in the previous step for all genes was determined, giving the normalization factor for each sample. The raw count value for each gene in each sample was then divided by this normalization factor, giving the normalized count value for each gene in each sample. Expression levels of genes are given as mean and SEM of DESeq2-normalized counts (*n* = 10).

### Statistical analysis

The data are reported as the mean ± SEM, unless stated otherwise. The statistical analysis was performed using Spearman's rank correlation coefficient (*r*) (IBM SPSS Statistics 23, IBM Corporation, NY, USA) and *t* tests (GraphPad Instat version 3.1 and GraphPad Prism version 5.02, GraphPad Software Inc., San Diego, CA, USA). A *p* value less than 0.05 was considered significant.

## Results

### RBP4 is present in the plasma and synovial fluid of the patients with osteoarthritis

**Fig. 1:**
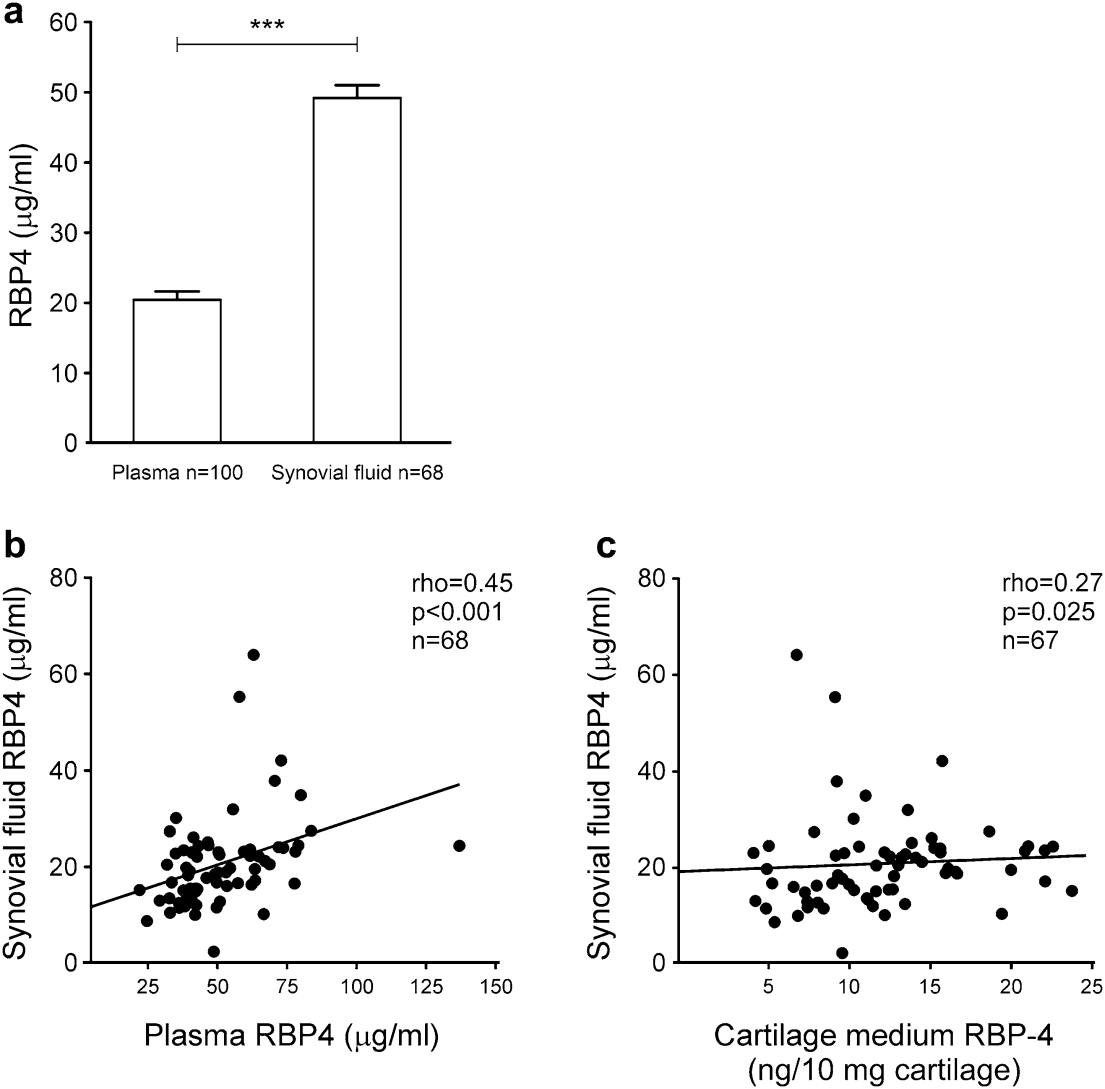
RBP4 is present in the synovial fluid from the OA patients, and the levels correlate positively with its concentration in the plasma and the cartilage culture medium. In **a** the results are expressed as the mean + SEM. *t* test was used to calculate statistical significance, ***p < 0.001. In **b** and **c** Spearman’s correlation analysis was used to detect any association

We measured the levels of RBP4 in the plasma and synovial fluid obtained from the OA patients. RBP4 was present at significant concentrations in the plasma and in the synovial fluid from the OA patients. RBP4 levels in plasma (49.2 ± 1.8 μg/ml) were higher than those in synovial fluid (20.4 ± 1.2 μg/ml, Fig. 1a), and there was a positive correlation between them (*r* = 0.45, *p* < 0.0001, Fig. 1b). The RBP4 levels in the synovial fluid also correlated with the amount of RBP4 released from the cultured cartilage obtained from the same patients (*r* = 0.27, *p* = 0.025, Fig. 1c).

### RBP4 correlates with adipokines and biomarkers in the cartilage from the patients with osteoarthritis

The cartilage samples from the OA patients released RBP4 protein into the culture medium (11.9 ± 0.5 ng/10 mg cartilage), and these levels correlated positively with the other adipokines measured: adiponectin (*r* = 0.54, *p* < 0.001), resistin (*r* = 0.38, *p* < 0.001), leptin (*r* = 0.29, *p* = 0.004) and adipsin (*r* = 0.27, *p* = 0.007). Interestingly, RBP4 also showed a positive correlation with the levels of MMP-1 (*r* = 0.26, *p* = 0.010), MMP-3 (*r* = 0.24, *p* = 0.017) and YKL-40 (*r* = 0.23, *p* = 0.025) released into the culture medium but did not correlate with BMI.

For the synovial fluid, no correlations between RBP4 and adipokines, MMP-1, MMP-3 or YKL-40 were found. The plasma RBP4 concentrations positively correlated with adipsin (*r* = 0.39, *p* < 0.0001) and MMP-3 (*r* = 0.25, *p* = 0.012).

### RBP4 is expressed in the primary human OA chondrocytes

As the results pointed to cartilage as a source of RBP4, we explored the expression of this adipokine and its receptors in chondrocytes from the OA patients by using RNA sequencing (RNA-Seq). As shown in Table 1, RBP4 was the most prominently expressed adipokine in the OA chondrocytes. We also observed that the receptors activated by RBP4, namely stimulated by retinoic acid gene homologue 6 (STRA6) and Toll-like receptor 4 (TLR4), were expressed in the OA chondrocytes (Table 1).

**Table 1:** Expression of retinol binding protein 4 (RBP4) and adipokines adipsin, adiponectin, leptin and resistin, as well as receptors activated by RBP4 in the OA chondrocytes as measured by RNA-Seq

Gene name	Expression	SEM
Adipokines		
Retinol binding protein 4	472.5	75.9
Adipsin	4.4	1.6
Adiponectin	0.8	0.5
Leptin	0.8	0.8
Resistin	< 0.1	< 0.1
Receptors activated by RBP4		
Stimulated by retinoic acid gene homologue 6	4.9	37.7
Toll-like receptor 4	234.1	1.9

The primary chondrocytes were isolated from knee cartilages of the OA patients (*n* = 10) undergoing knee replacement surgery and cultured for 24 h. Expression levels of genes are given as mean and SEM of DESeq2-normalized counts

## Discussion

RBP4 is a retinol transport protein in blood that is prevalently expressed in the liver but is also highly present in adipose tissue [6, 7]. Because of the close correlation between obesity, MetS and OA [6, 9–11, 14, 31], we analysed here, for the first time, the potential associations of RBP4 into the pathogenesis of OA.

To date, there have been no studies on the RBP4 production in joint tissues. Here, we showed that cultured cartilage released RBP4. Furthermore, these RBP4 levels correlated positively with the other adipokines implicated in OA pathogenesis, i.e., adiponectin [24, 32–34], resistin [35, 36], leptin [23, 37–41] and adipsin [41, 42]. All these adipokines can be found in the synovial fluid of OA joints. Adipokines are produced, e.g., in adipose tissue, and from the circulation, they likely diffuse into the joint. They can also be produced intra-articularly, and the expression of leptin and adiponectin mRNA/protein has been previously detected in OA cartilage and chondrocytes [33, 43]. In the expression analysis presented here, human OA chondrocytes also expressed adipsin and RBP4, and in fact, RBP4 was the most prominently expressed of the measured adipokines in the OA chondrocytes (Table 1).

RBP4 acts as an immunomodulatory adipocytokine. Kahn et al. reported that RBP4 induces the secretion of proinflammatory cytokines in mouse dendritic cells and macrophages, causing Th1 polarization and proliferation in vivo [44]. RBP4 also activates APCs (antigen-presenting cells) in adipose tissue through the JNK pathway, promoting adipose tissue inflammation and systemic insulin resistance [44]. It has been demonstrated that immune cells play a role in the pathogenesis of OA and that OA synovium contains T cells and increased levels of Th1 cytokines [34, 45–48]. Therefore, it is reasonable to suggest that, as with leptin [49], RBP4 found within the joint could modulate the inflammatory milieu in a manner that promotes cartilage damage in OA.

In the present study, we show that OA synovial fluid contains RBP4 and that there is a significant positive correlation between the RBP4 levels in the OA synovial fluid and the cartilage culture medium from the same patients. Regarding the synovial fluid, we did not find any correlations between the RBP4 levels and the OA biomarkers. This finding is likely due to the contribution by other joint tissues than cartilage to the content of the synovial fluid, but this possibility was not explored in the present study. Instead, the findings point to cartilage as a significant target tissue that bears the destructive effects of RBP4. OA chondrocytes express STRA6 and TLR4, receptors known to be activated by RBP4 [17–21]. The positive correlation between the cartilage-derived RBP4 and the degrading enzymes MMP-1 and MMP-3 and the OA-related inflammatory marker YKL-40 could be due to the induction of these factors by the RBP4 in the cartilage, likely through the activation of the TLR4 receptor, which has been suggested to mediate some of the pro-inflammatory effects induced by RBP4 [19–21, 50]. Recent studies of other tissues have shown that RBP4 has the ability to stimulate MMP release and that the knocked-down RBP4 suppressed the expression of the matrix metalloproteinases [51, 52]. Based on these studies and the findings presented in this work, RBP4 could have similar effects in cartilage.

We also measured the levels of RBP4 in the plasma from the OA patients. These levels correlated positively with adipsin, an adipokine recently demonstrated as a factor that contributes to cartilage degradation in osteoarthritis [41, 42]. Moreover, for plasma, we found a positive correlation between RBP4 concentration and MMP-3, a classic biomarker of OA [53, 54]. The plasma from the OA patients contained more RBP4 than the SF, and we observed a positive correlation between the RBP4 concentration in these two compartments. Therefore, it is likely that, in addition to local production, RBP4 produced by the liver and adipose tissue diffuses into the joint from the circulation.

Several studies reported an increased concentration of RBP4 in obesity [9, 55–57] and in conditions related to obesity complications, including metabolic syndrome [58, 59], diabetes [9, 10, 60] and cardiovascular diseases [61–63]. Reported RBP4 levels normally range between 20 and 40 µg/ml in non-OA study populations [9–11], while RBP4 concentrations were at 50 µg/ml-level in OA patients in the present study. Direct comparisons are not advisable because of differences in the populations and the methods used. However, it is noteworthy that obesity is a major risk factor for OA, and OA is increasingly regarded as the fifth clinical feature of metabolic syndrome, which makes the adipokine RBP4 a potential novel factor linking the metabolic state, inflammation and articular degradation in OA.

In conclusion, our data demonstrated, for the first time, that RBP4 is a prominently expressed adipokine in OA chondrocytes and is present in synovial fluid and plasma from OA patients at considerable μg/ml levels. RBP4 was also found to be associated with increased levels of adipokines and matrix metalloproteinases MMP-1 and MMP-3.

The results suggest a role for RBP4 in the pathogenesis of OA and as a potential target for disease-modifying drugs for the treatment of OA, which encourages additional studies to reveal the more detailed role of RBP4 in the pathogenesis and pathology of OA. Further studies in large cohorts of patients and in cells and joint tissues are needed to confirm the implications of RBP4 in OA.
